# Retinal image quality in myopic children undergoing orthokeratology alone or combined with 0.01% atropine

**DOI:** 10.1186/s40662-023-00339-0

**Published:** 2023-06-01

**Authors:** Qi Tan, Pauline Cho, Alex L. K. Ng, George P. M. Cheng, Victor C. P. Woo, Stephen J. Vincent

**Affiliations:** 1 School of Optometry, The Hong Kong Polytechnic University, Hung Hom, Kowloon, Hong Kong SAR, China; 2 Department of Ophthalmology, The University of Hong Kong, Hong Kong SAR, China; 3 Hong Kong Ophthalmic Associates, Hong Kong SAR, China; 4 Hong Kong Laser Eye Centre, Hong Kong SAR, China; 5 Contact Lens and Visual Optics Laboratory, Centre for Vision and Eye Research, School of Optometry and Vision Science, Queensland University of Technology, Brisbane, Australia

**Keywords:** Combined treatment, Orthokeratology, 0.01% atropine, Myopia, Retinal image quality, Ocular aberrations

## Abstract

**Background:**

The retinal image quality derived from lower-order (LOA) and higher-order aberrations (HOA) for fixed 3-mm and photopic pupil diameters, in children undergoing combined 0.01% atropine and orthokeratology (AOK) versus those receiving orthokeratology alone (OK) over two years was evaluated.

**Methods:**

The visual Strehl ratio based on the optical transfer function (VSOTF), derived from 2nd- to 4th-order terms (LOA and HOA combined), 2nd-order terms (LOA only), and 3rd- to 4th-order terms (HOA only) for fixed 3-mm and natural photopic pupil diameters, was compared between the two treatment groups. The individual Zernike coefficients for a fixed 3-mm pupil size of 2nd- to 4th-orders, root mean square (RMS) of LOA (${Z}_{2}^{0}$, ${Z}_{2}^{-2}$, and ${Z}_{2}^{2}$ combined), HOA (3rd to 4th orders inclusive), and Coma (${Z}_{3}^{-1}{\mathrm{and }Z}_{3}^{1}$ combined) were also compared between the two groups.

**Results:**

Right eye data of 33 AOK and 35 OK participants were analysed. Under photopic conditions, significantly lower VSOTF based on HOA only was observed in the AOK group compared with that in the OK group at all post-treatment visits (all *P* < 0.05); however, interactions between HOA and LOA resulted in comparable overall retinal image quality (i.e., VSOTF based on LOA and HOA combined) between the two groups at all visits (all *P* > 0.05). For a fixed 3-mm pupil size, the VSOTF based on HOA only, LOA only, or HOA and LOA combined, were not different between the two groups (all *P* > 0.05). AOK participants had slower axial elongation (mean ± SD, 0.17 ± 0.19 mm vs. 0.35 ± 0.20 mm, *P* < 0.001), a larger photopic pupil size (4.05 ± 0.61 mm vs. 3.43 ± 0.41 mm, *P* < 0.001) than OK participants, over two years.

**Conclusions:**

HOA profile related to an enlarged pupil size may provide visual signal influencing eye growth in the AOK group.

## Background

Animal studies across a wide range of species have shown that altering the visual experience during early life influences short and long-term eye growth [1–5]. Based on these results, retinal image quality has been hypothesized as a potential factor that could affect axial eye growth in children [6], due to either the natural [7] or altered [8] optics of the eye. Of the optical interventions used to retard axial elongation in myopic children, orthokeratology (ortho-k) has typically shown greater efficacy [9], with an average of 43% to 63% less axial elongation over two years, compared to children wearing single-vision spectacles [10–15] or soft contact lenses [16]. Following ortho-k, lower-order aberrations (LOA, defocus, and astigmatism), are largely corrected across the central field [10–16]. However, the reshaped cornea significantly alters the higher-order aberration (HOA) profile, as the root mean square (RMS) of HOA increased from 0.18 to 0.49 µm for a 4-mm pupil [17], and from 0.27 to 0.69 µm for a 6-mm pupil [18] (i.e., almost a tripling of total HOA for 4–6 mm pupil diameters); which is sustained over long-term ortho-k treatment [19, 20]. In children undergoing ortho-k, less axial elongation has been associated with greater increases in HOA RMS [19, 20], comatic aberrations RMS (e.g. $${Z}_{3}^{-1}{, Z}_{3}^{1}{, Z}_{5}^{-1},$$ and $${Z}_{5}^{1}$$ combined) [20, 21], and a positive shift in primary spherical aberration ($${Z}_{4}^{0})$$[19].

Of note, in previous ortho-k studies, the predominant optical metrics considered were HOA RMS or individual Zernike coefficients, but not retinal image quality [19–21]. In addition, many ortho-k studies have examined HOA using a fixed pupil diameter (typically 4–6 mm) across all participants [19–21]. However, throughout the day the eye is typically exposed to photopic levels of ambient lighting resulting in a constricted pupil diameter of typically less than 4 mm (on average 3.72 mm in children aged 4–12 years and 3.94 mm in children aged 6–12 years) [22, 23]. Thus, an analysis of retinal image quality based on photopic pupil sizes may be more relevant to understand the visual quality of children undergoing ortho-k during typical daytime activities.

Several studies have now investigated the effect of a combination treatment using low concentration (0.01%) atropine with ortho-k (AOK) to retard axial elongation [24–26], assuming that atropine may enhance the optical effect of ortho-k through pupil dilation or by directly influencing muscarinic receptors in the eye that regulate eye growth [27]. Different mechanisms of action are believed to be involved, with ortho-k potentially slowing axial elongation due to alterations in peripheral refraction [28, 29] or HOA [8], while atropine exerts effects on anti-muscarinic receptors of the retina and sclera to slow myopia progression [30, 31]. An improved effect in retarding axial elongation of AOK treatment compared to ortho-k alone (OK) was observed in the atropine combined with ortho-k study [26], and in a subgroup of myopic children with a baseline spherical equivalent refraction (SER) less than 3.00 D, over two years [24]. An analysis of ocular HOA after six months of AOK treatment suggested that the improved retardation of axial elongation was potentially related to the optical effect of an enlarged photopic pupil [32]. The current study aimed to compare the retinal image quality (i.e., the visual Strehl ratio based on the optical transfer function, VSOTF) and aberration metrics derived LOA and HOA for a fixed 3-mm pupil size and retinal image quality for photopic pupil sizes in children undergoing AOK and OK treatment over two years.

## Methods

### Participants and materials

The design of the current study (ClinicalTrials.gov: NCT02955927) has been described previously [33]. Participants were recruited via advertisements in local newspapers or by word of mouth, and those who passed a subsequent phone screening were invited to attend a screening examination to assess their eligibility. In brief, children of Chinese ethnicity aged 6 to less than 11 years with normal ocular health other than 1.00–4.00 D of myopia, ≤ 2.50 D astigmatism, and < 1.00 D difference in SER between the two eyes, no history of myopia control treatment, were randomized into either the treatment AOK or OK group in a 1:1 ratio. The sample size calculation was based on the within group standard deviation (SD) of 0.25 mm from the retardation of myopia in orthokeratology (ROMIO) study [11], to achieve 80% power to detect a minimum difference of 0.18 mm in axial length over two years with a 5% level of significance. At least 48 participants (24 in each group) were required at completion. AOK treatment involved the application of one drop of preservative-free 0.01% atropine (Aseptic Innovative Medicine Co., Ltd., Taiwan, China) in each eye, 10 min before nightly wear of ortho-k lenses (KATT BE Free Lens, Precision Technology Services, Vancouver, B.C., Canada), while OK treatment only involved wearing the same design ortho-k lenses nightly. Parameters of ortho-k lenses were calculated using the Eye Care Practitioner Software (Precision Technology Services, Vancouver, B.C., Canada), based on corneal topography (E300, Medmont, Australia), non-cycloplegic manifest refraction, and the horizontal visible iris diameter. If a difference of more than 30 μm in the corneal sag between the horizontal and vertical meridians was observed for an 8-mm chord diameter, a toric lens design was used; otherwise, a spherical design was used per the manufacturer’s recommendation. Lens refitting was performed if ≤ − 0.50 D non-cycloplegic residual SER was found at post-treatment visits with continual lens wear of at least seven nights, or if moderate to severe decentration of a lens (> 1 mm) was observed. Lens was yearly replaced if no refit would be indicated. Complimentary contact lens solutions were provided to participants to ensure their compliance with solution replacement monthly. Each vial of single-dose 0.01% atropine eye drops (0.5 mL per vial) contains 0.05 mg atropine sulphate compounded with saline. Compliance was assessed by calculating the rate of using atropine eye drops in the AOK group (total numbers of returned empty vials/total number of days during the study), as well as the rate of ortho-k lens wear (total number of nights with lens wear/total number of days during the study) in all participants. Ethical approval was obtained from the Human Subject Ethics Subcommittee of the School of Optometry of the Hong Kong Polytechnic University (PolyU) (Reference No. HSEARS20160406005) and the Institutional Review Board of the University of Hong Kong/Hospital Authority Hong Kong West Cluster (Reference No. UW 16_404). A certificate for the clinical trial/medicinal test was obtained from the Pharmacy and Poison Board, Department of Health of Hong Kong (Reference No. PR/CT 0118/2016(AL)). Assent and consent were provided by children and parents, respectively, before their participation. All participants were required to attend cycloplegic examinations (data collection visits) every six months after commencement of the treatment at the Optometry Clinic of the School of Optometry of PolyU. All procedures followed the tenets of the Declaration of Helsinki.

### Examination procedures

For each participant, data collection at subsequent visits was carried out within ± 2 h of the measurement time of the baseline visit at 6-monthly intervals. Manifest subjective refractive error was measured by an unmasked examiner, before and after cycloplegia using a trial frame, following the principle of maximum plus for maximum visual acuity (VA). Unaided VA (UVA) and best-corrected VA (BCVA) were measured using high contrast (100%) Early Treatment Diabetic Retinopathy Study charts (Precision Vision, La Salle, Illinois, USA) under normal room lighting at a 4-m distance. Pupil size was measured by the unmasked examiner who was not involved in the follow-up of participants, using the OPD-Scan III (Nidek, Gamagori, Japan) with an internal light source, under mesopic illuminance (3.5 lx, approximately 36 Trolands for a mean mesopic entrance pupil diameter of 6.43 mm across all visits [34]), followed by photopic illuminance (125.6 lx, approximately 318 Trolands for a for a mean photopic entrance pupil diameter of 3.18 mm entrance pupil diameter across all visits [34]) in a closed dark room with the lights off (0 lx). For each participant, retinal illuminance was calculated using a method described by Thibos et al. [34], based on the average photopic pupil size across all post-treatment visits. Participants were required to fixate on the internal instrument target and were fogged to relax their accommodation during the examination. The first three measurements with a difference of less than 0.50 mm were averaged for analyses. With the optimal distance refraction in place, the amplitude of accommodation was measured three times, using a Royal Air Force Rule (Harlow, Essex, UK) (push up method) for each eye, which was later averaged for analyses.

The procedures for measuring monochromatic ocular aberrations using a Complete Ophthalmic Analysis System (COAS) (Wave-front Sciences Ltd., New Mexico, USA) have been described in detail previously [32]. The COAS utilises a 33 × 44 lenslet array with each lenslet 144 μm in diameter. Since the pupil magnification factor is about 0.685, the lenslet array samples the exiting wavefront every 210 μm in the pupil plane (i.e., 600 samples for a 6-mm pupil diameter) [35]. Under non-cycloplegic conditions, aberrations were measured while participants fixated an external Maltese monocularly cross target illuminated by an incandescent lamp (5.3 lx), viewed through a beam splitter and Badal lens in a closed dark room. To provide a 0.00 D stimulus to accommodation during the examination, the position of the target was altered accordingly by compensating for the participants’ distance SER. The first five wavefront measurements without a blink (each consisting of 25 measurements captured within two seconds) were exported for each eye. The exported data were fitted with a Zernike polynomial expansion up to the 6th-order based on a 6-mm pupil diameter. Potential measurement artefacts, within the 125 measurements captured at each visit for each participant, indicative of fluctuating accommodation, were removed using customized software (i.e., a difference in pupil diameter >  ± 0.50 mm, or difference in defocus >  ± 0.50 D from the sample median). The measured coefficients for a 6-mm pupil diameter were then re-scaled down to the natural photopic pupil sizes measured with the OPD-Scan III and a fixed 3-mm pupil size (mean ± SD photopic pupil size was 3.45 ± 0.54 mm in all participants across all visits), respectively, following the method described by Schwiegerling [36]. The re-scaled coefficients were provided up to the 6th-order, but analysis was only performed up to the 4th-order, as coefficients of the 5th- and 6th-orders were small for 3–4 mm pupil diameters [37], which were suggested to be merely instrument noise measured using the COAS instrument [38]. Based on the individual Zernike coefficients for a fixed 3-mm pupil size of 2nd- to 4th-orders (inclusive), RMS of LOA (${Z}_{2}^{0}$, ${Z}_{2}^{-2}$, and ${Z}_{2}^{2}$ combined), HOA (3rd- to 4th-orders inclusive), and Coma (${Z}_{3}^{-1}{\mathrm{ and }Z} _{3}^{1}$ combined) were calculated, respectively. Retinal image quality for distance viewing was quantified using the VSOTF, which was derived from 2nd- to 4th-order terms (LOA and HOA combined), 2nd-order terms (LOA only), and 3rd- to 4th-order terms (HOA only), for a fixed 3-mm pupil size and the natural photopic pupil sizes for each participant at each visit, according to a method described previously [39]. VSOTF was used for description of the retinal image quality due to its high correlation with visual acuity [40]; and with a value range from 0 to 1, the higher the VSOTF, the better the retinal image quality [41]. Axial length was measured with the IOLMaster (Carl Zeiss Meditec AG, Jena, Germany) by a masked examiner at least 30 min after cycloplegia using two drops of 1% cyclopentolate five minutes apart. The composite readings, determined from five readings with a maximum difference of 0.02 mm and signal-to-noise ratio above five, were used for analyses.

### Statistical analyses

As HOA tend to be mirror-symmetric between the left and right eyes [42], only data from the right eye was included in the analyses. Statistical analyses were performed using SPSS version 25 (IBM Corp., Armonk, NY, USA). The normality of data, such as age, axial length, retinal illuminance, individual aberration metrics for a 3-mm pupil (RMS of LOA, HOA, and Coma, and coefficients of individual Zernike terms of 2nd- to 4th-orders), and timing of the baseline visit were assessed using the Kolmogorov-Smirnov test. Crosstab analysis was used to compare the sex distribution between groups. After confirming the normality of baseline data (age, BCVA, and axial length), retinal illuminance across all post-treatment visits, and timing of the data-collection visits over 24 h (for all participants and those visiting in the afternoon), an unpaired t-test was used to compare these data between the two groups. Other normally distributed data, including axial elongation, pupil size, the amplitude of accommodation, non-cycloplegic SER and UVA, individual aberration metrics for a 3-mm pupil (RMS of LOA, HOA, and Coma, and coefficients of individual Zernike terms of 2nd- to 3th-orders), and VSOTF for a 3-mm pupil size and natural photopic pupil sizes over two years were compared using a two-way repeated-measures analyses of variance (RM ANOVA), with Bonferroni-corrected post hoc comparisons to examine the between-group and between-visit differences, and their interactions, where appropriate. As the coefficients of individual Zernike terms of 4th-order for a 3-mm pupil and timing of data-collection visits for participants visiting in the morning were not normally distributed, the Mann-Whitney U test was used to compare these data between the two groups, and a Bonferroni-adjusted *P* value of less than 0.01 (0.05/5) was considered statistically significant, where appropriate.

## Results

### Demographics

**Fig. 1:**
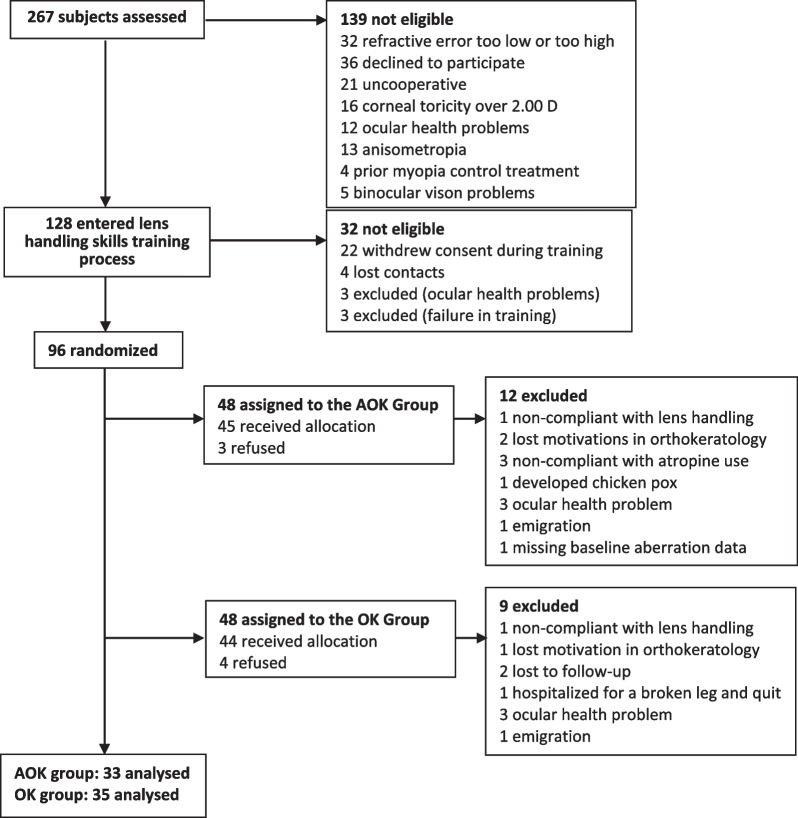
Flow chart showing participants recruitment and dropouts. AOK, combined atropine with orthokeratology; OK, orthokeratology alone

Of the total of 89 participants (45 AOK and 44 OK) who commenced the treatment, 11 AOK and nine OK participants were excluded at different stages of the study for various reasons. Of the 69 participants (34 AOK and 35 OK) who completed the two-year study, one AOK participant was excluded from the analysis due to missing baseline aberration data (Fig. 1). Analyses were performed for 68 participants, with the mean rate for lens wear at 93% (95% confidence interval: 92%–94%) for both groups and the same rate and confidence intervals for application of atropine eye drops. The demographics and baseline data showed no significant differences in sex, age, BCVA, axial length (all *P* > 0.05, Table 1), pupil size (mesopic and photopic), non-cycloplegic SER, and the amplitude of accommodation (all *P* > 0.05, Table 2) between the AOK and OK groups. Over two years, data of 11 AOK and 12 OK participants were taken in the morning, while data of 22 AOK and 23 OK participants were taken in the afternoon; the timing over 24 h of the baseline visit (all *P* > 0.05, Table 1) and post-treatment visits at 6-month intervals was similar for the two groups (all *P* > 0.05).

**Table 1:** Baseline demographic data and timing of baseline visit. Data are presented as mean ± standard deviation

Characteristic	AOK	OK	*P*
Number of participants	33	35	
Sex (female/male)	15/18	15/20	0.44
Age (years)	9.3 ± 1.0	9.1 ± 1.2	0.50
BCVA (logMAR)	− 0.04 ± 0.05	− 0.03 ± 0.05	0.42
Axial length (mm)	24.57 ± 0.71	24.50 ± 0.92	0.73
Timing of baseline visit (hours)	13:54 ± 3:06	13:42 ± 3:30	0.83

Visit in the morning	AOK	OK	*P*
Number of participants	11	12	–
Timing of baseline visit (hours)*	9:00 (9:00–12:00)	9:36 (8:00–10:54)	0.99

Visit in the afternoon	AOK	OK	*P*
Number of participants	22	23	–
Timing of baseline visit (hours)	15:54 ± 1:18	16:00 ± 1:36	0.89

*AOK *= combined 0.01% atropine and orthokeratology; *OK *= orthokeratology alone

*median (range) is presented as data is not normal

**Table 2:** Summary of results of combined 0.01% atropine and orthokeratology (AOK) and orthokeratology alone (OK) groups at each visit

Metric	Group	Mean ± SD					Interaction (F, *P*)
		Baseline	6-month	12-month	18-month	24-month	Group x visit
Axial elongation (mm)	AOK	–	–0.02 ± 0.10	0.06 ± 0.14	0.11 ± 0.17	0.17 ± 0.19	4.76, **0.014**
	OK	–	0.07 ± 0.08	0.18 ± 0.15	0.25 ± 0.18	0.35 ± 0.20	
Difference [SE]		–	0.09 [0.02]	0.11 [0.04]	0.14 [0.04]	0.18 [0.05]	
*P*^‡^		–	** < 0.001**	**0.002**	**0.001**	** < 0.001**	
Photopic pupil (mm)	AOK	3.25 ± 0.31	3.81 ± 0.45	3.88 ± 0.63	3.91 ± 0.59	4.05 ± 0.61	14.91, < **0.001**
	OK	3.20 ± 0.32	3.16 ± 0.33	3.22 ± 0.35	3.30 ± 0.42	3.43 ± 0.41	
Difference [SE]		0.05 [0.08]	0.64 [0.10]	0.66 [0.12]	0.61 [0.12]	0.62 [0.13]	
*P*^‡^		0.49	** < 0.001**	** < 0.001**	** < 0.001**	** < 0.001**	
Mesopic pupil (mm)	AOK	6.51 ± 0.86	7.15 ± 0.64	7.12 ± 0.62	7.18 ± 0.59	7.21 ± 0.54	10.35, < **0.001**
	OK	6.61 ± 0.82	6.72 ± 0.78	6.74 ± 0.83	6.72 ± 0.81	6.92 ± 0.78	
Difference [SE]		0.10 [0.20]	0.43 [0.17]	0.38 [0.18]	0.46 [0.17]	0.29 [0.16]	
*P*^‡^		0.09					
Amplitude of accommodation (D)	AOK	13.5 ± 1.9	11.9 ± 1.5	12.2 ± 1.3	11.4 ± 1.3	11.2 ± 1.5	2.40, 0.08
	OK	12.8 ± 2.1	12.2 ± 1.4	12.1 ± 1.1	11.7 ± 1.2	11.6 ± 1.1	
Difference [SE]		0.7 [0.5]	0.2 [0.4]	0.1 [0.3]	0.3 [0.3]	0.4 [0.3]	
*P*^‡^		0.91					
Non-cycloplegic SER (D)	AOK	–2.65 ± 0.92	0.28 ± 0.45	0.22 ± 0.44	0.21 ± 0.40	0.05 ± 0.44	0.59, 0.57
	OK	–2.88 ± 0.98	–0.06 ± 0.46	–0.01 ± 0.44	–0.20 ± 0.45	–0.12 ± 0.44	
Difference [SE]		0.22 [0.23]	0.34 [0.11]	0.22 [0.11]	0.41 [0.10]	0.17 [0.11]	
*P*^‡^		0.34	**0.003**	**0.039**	** < 0.001**	0.12	
Non-cycloplegic unaided visual acuity (logMAR)	AOK	0.98 ± 0.41	–0.02 ± 0.08	–0.02 ± 0.09	–0.01 ± 0.06	0.00 ± 0.07	0.19, 0.72
	OK	0.98 ± 0.42	0.01 ± 0.11	0.01 ± 0.08	0.03 ± 0.11	0.03 ± 0.09	
Difference [SE]		0.01 [0.10]	0.03 [0.02]	0.03 [0.02]	0.04 [0.02]	0.03 [0.02]	
*P*^‡^		0.36					

*SER *= spherical equivalent refraction; *SD *= standard deviation; *SE *= standard error

*P*: probability value of two-way RM-ANOVA indicating the interaction between group and visit, with *P*^‡^ for post hoc test showing differences between groups at each visit. Bold typeface values indicate statistical significance

### Pupil size, retinal illuminance, the amplitude of accommodation, non-cycloplegic SER and UVA over two years

A significant group by visit interaction was observed for both photopic and mesopic pupil diameters (both *P* < 0.001), with post hoc comparisons indicating a significant between-group difference in the photopic (all *P* < 0.001), but not the mesopic pupil size (*P* = 0.09), at all post-treatment visits (Table 2). For the photopic pupil sizes, the mean ± SD retinal illuminance averaged across all post-treatment visits was significantly higher in the AOK group than that of the OK group (mean ± SD, AOK *vs.* OK, 459.3 ± 107.5 Trolands *vs.* 338.7 ± 65.4 Trolands, *P* < 0.001). There was no main effect of group or a group by visit interaction for the amplitude of accommodation (both *P* > 0.05), indicating no significant between-group difference in this parameter over two years. Given that residual myopic SER was over 0.50 D at follow-up visits, 14 OK participants were refitted with 20 lenses over two years, whereas four AOK participants were refitted with four lenses. After the two-year treatment, non-cycloplegic SER was not significantly different between the two groups (*P* = 0.12, Table 2). For non-cycloplegic UVA, there was no main effect of group or a group by visit interaction (both *P* > 0.05, Table 2), indicating no significant between-group difference in this parameter over two years.

### Comparison of aberrations and retinal image quality for a fixed 3-mm pupil size and photopic pupil sizes

**Fig. 2:**
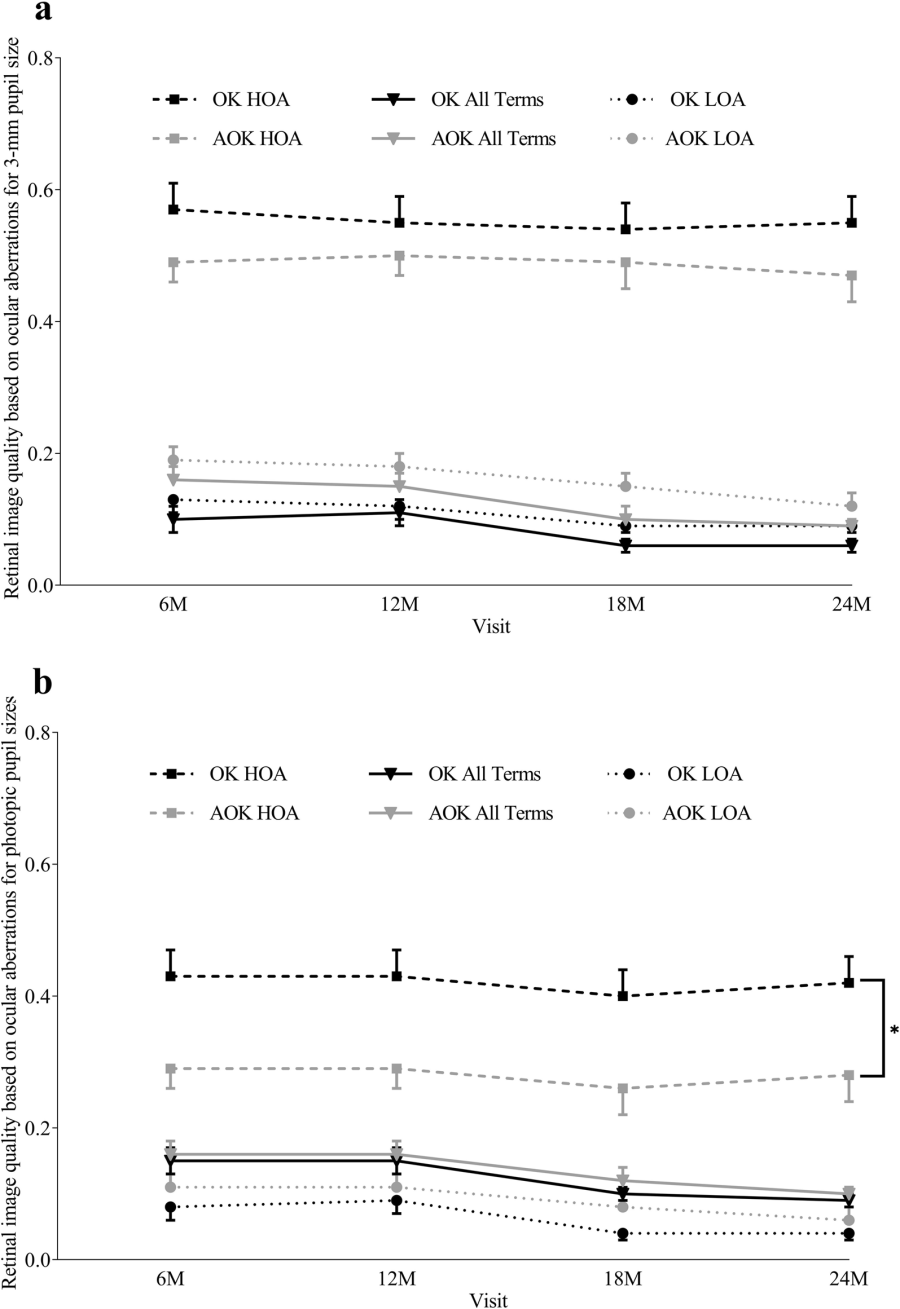
Retinal image quality calculated from aberration terms over time for the two groups.** a** 3-mm pupil size; **b** Natural photopic pupil sizes. Error bars indicating the standard error. AOK, combined 0.01% atropine and orthokeratology; OK, orthokeratology alone; HOA, higher-order aberrations terms (3rd- to 4th-orders inclusive orders); LOA, lower-order aberrations terms (2nd-order); OK HOA, mean visual Strehl ratio derived from HOA only in the OK group (n = 35); OK LOA, mean visual Strehl ratio derived from LOA only in the OK group (n = 35); OK All Terms, mean visual Strehl ratio derived from HOA and LOA combined in the OK group (n = 35); AOK HOA, mean visual Strehl ratio derived from HOA only in the AOK group (n = 33); AOK LOA, mean visual Strehl ratio derived from LOA only in the AOK group (n = 33); AOK All Terms, mean visual Strehl ratio derived from all Zernike terms HOA and LOA combined in the AOK group (n = 33); M, months; *indicating significant difference at all post-treatment visits

For a fixed 3-mm pupil, there were no significant differences in any RMS (LOA, HOA, and Coma), individual Zernike coefficients of LOA and HOA, or retinal image quality (i.e., the VSOTF based on LOA and HOA combined, VSOTF based on LOA only, VSOTF based on HOA only) between the two groups at all visits (all *P* > 0.05) (Fig. 2a). There were no significant within-group differences in LOA RMS, HOA RMS, or retinal image quality in each group (i.e., the VSOTF based on LOA and HOA combined, VSOTF based on LOA only, VSOTF based on HOA only) between any post-treatment visits (all *P* > 0.05). In the AOK group, mean ± SD value of LOA RMS were 1.14 ± 0.38 µm at the baseline visit and 0.31 ± 0.23 µm at the 24-month visit; in the OK group, LOA RMS were 1.06 ± 0.37 µm at the baseline visit and 0.40 ± 0.22 µm at the 24-month visit. Mean ± SD of HOA RMS increased from 0.06 ± 0.06 µm and 0.04 ± 0.02 µm at the baseline visit, to 0.11 ± 0.05 µm and 0.09 ± 0.05 µm at the 24-month visit, in the AOK and OK groups, respectively.

For photopic pupil sizes, no significant differences in the VSOTF based on LOA and HOA combined, and VSOTF based on LOA only, were observed between the two groups at all visits (all *P* > 0.05); however, the VSOTF based on HOA only was significantly higher in the OK group than the AOK group at all post-treatment visits (all *P* < 0.05) (Fig. 2b). In each group, there was no significant differences in retinal image quality (i.e., VSOTF based on LOA and HOA combined, VSOTF based on LOA only, VSOTF based on HOA only) between any post-treatment visits (all *P* > 0.05).

### Axial elongation

AOK participants had less axial elongation than those in the OK group over two years (mean ± SD, 0.17 ± 0.19 mm *vs.* 0.35 ± 0.20 mm, *P* < 0.001). A significant group by visit interaction was observed for axial elongation (*P* = 0.014), with post hoc analyses indicating that the axial elongation was significantly greater in the OK group than that of the AOK group at all post-treatment visits (all *P* < 0.01).

## Discussion

Retinal image quality in terms of VSOTF was reported to get worse during accommodation in non-myopic children [43] and progressing myopes wearing best-corrected spectacles [44]. It was also assessed longitudinally in emmetropes who became myopes five years after initial assessment [45]. However, decreased retinal image quality was not associated with refractive error development [45]. Long-term studies for retinal image quality in children undergoing myopia control treatment are lacking. The current study was the first to assess and compare the retinal image quality during distance viewing (relaxed accommodation through a Badal system) in children undergoing treatment of AOK and OK to retard axial elongation over two years. The major findings of our study were that, for photopic pupil sizes, retinal image quality based on HOA only was significantly reduced in the AOK group compared to the OK group at all post-treatment visits (all *P* < 0.05); however, the interaction between HOA and LOA terms yielded comparable overall retinal image quality (i.e., based on HOA and LOA combined). After the two-year treatment, axial elongation of AOK participants was 0.18 mm significantly less than those in the OK group, indicating that AOK treatment had an improved long-term effect in retarding axial elongation over OK treatment. After treatment, mean of the non-cycloplegic SER was positive in the AOK group, while negative values were presented in the OK group (Table 2); the significant between-group differences in the non-cycloplegic SER (except at 24-month visit), may explain why the interaction of LOA and HOA produced a non-significant difference in the quality of the retinal image between the two groups. But based on VSOTF analysis derived from HOA, it is still possible that HOA profile related to an enlarged pupil size in the AOK group may provide visual signal influencing eye growth, given the reduced axial elongation in this group.

Here, to maintain good UVA during the day, lenses were refitted if the participant’s residual myopic SER was 0.50 D or more [46]. In line with this protocol, there was a higher rate of lens refits in the OK group than that in the AOK group to compensate for the residual myopic SER that highly likely resulted from axial elongation. Therefore, it is reasonable to observe that non-cycloplegics were not different between the two groups after two years of treatment (Table 2). For the photopic pupil sizes, the mean difference of 0.62 mm between the two groups was clinically significant, as 0.25 mm is the degree of accuracy for photopic pupil size measurements using OPD-Scan III [47]. Notably, measurements of photopic pupil sizes were acquired in the morning or afternoon, and it is possible that the pupil dilation resulting from use of 0.01% atropine may wear off during the daytime. For the subgroup of participants (22 AOK and 23 OK) who were examined in the afternoon, a larger post-treatment photopic pupil size was still observed in the AOK group than in the OK group over two years (mean ± SD, 3.92 ± 0.57 mm *vs.* 3.31 ± 0.41 mm, *P* < 0.001), whereas their baseline photopic pupil size was similar (*P* = 0.58). This indicates that the between-group difference in the photopic pupil size was maintained over a substantial proportion of waking hours. Over two years, there was a mean reduction of 2.3 D and 1.2 D in accommodation in the AOK and OK groups, respectively. However, baseline accommodation was robust (Table 1), and the reduction in accommodation did not result in a different profile of accommodation between the two groups (*P* > 0.05, Table 2).

The use of atropine can alter the ocular HOA profile due to pupil dilation [6] and/or the paralysis of accommodation [48]. Also, ocular aberrations may change following a lens refit, particularly since there was a higher lens refit rate in the OK group. In the current study, for a fixed 3-mm pupil diameter, no between-group differences were observed for individual Zernike coefficients of all orders (including LOA, HOA, and particularly defocus), LOA RMS, and HOA RMS. That is, when a fixed 3-mm pupil diameter was considered, where the effect of the pupil size was controlled, the use of 0.01% atropine in the AOK group or a higher rate of lens refit in the OK group, did not cause a difference in the aberration profile between the two groups. Moreover, LOA was not fully corrected in ortho-k treated eyes, as for a fixed 3-mm pupil, the mean ± SD defocus (${Z}_{2}^{0}$) was reduced by 73% and 63% in the AOK and OK groups, respectively, while HOA RMS doubled in both groups. Following reductions in LOA and increases in HOA after treatment, the ratio of LOA to HOA (RMS) was changed significantly from approximately 30:1 for a 3-mm pupil pre-treatment in each group, to 3:1 in the AOK group and 4:1 in the OK group, after two-year treatment.

Due to the use of 0.01% atropine, the mean photopic pupil size was 0.62 mm larger in the AOK group than those in the OK group after two years of treatment. Of note, the magnitude of HOA is elevated with increased pupil size [37], and elevated HOA may influence the quality of the retinal image. In line with this speculation, for photopic pupil sizes, retinal image quality derived from HOA only was reduced in the AOK group compared with that in the OK group at all post-treatment visits (all *P* < 0.05). However, the overall retinal image quality was not different between the two groups at all post-treatment visits (all *P* > 0.05), suggesting interactions between HOA and LOA terms. One limitation of our study is that retinal image quality and aberration profile was described only during relaxed accommodation. Simulation analyses suggest that retinal image quality during accommodation can be affected by a combination of HOA and LOA terms (i.e., ${Z}_{4}^{0}$ and defocus) [49–51], and this interaction may provide a directional cue to the retina and consequently alter axial eye growth [52]. In addition, compared with the OK group, there was reduced VSOTF based on HOA only and less axial elongation in the AOK group, suggesting that there may be a link between potential visual signal related to HOA and axial elongation. Results obtained in this study only indicate that a similar profile of retinal image quality in the AOK and OK groups during distance viewing, either for a fixed 3-mm pupil or for the photopic pupil sizes. However, the retinal image quality during accommodation in both groups are unknown. Future studies are warranted to investigate whether retinal image quality and the aberration profile at different accommodative demands are linked with axial eye growth in children undergoing AOK or OK treatment. Also, retinal image quality in the current study was based on on-axis ocular aberrations measured with COAS, but ocular aberrations may also be presented off-axis and affect the peripheral image quality [49]. Further studies concerning the off-axis aberration along with the peripheral defocus are warranted.

## Conclusion

The use of 0.01% atropine nightly in conjunction with ortho-k resulted in slower axial elongation compared to single treatment with ortho-k. Despite increased photopic pupil diameters induced with the use of 0.01% atropine, retinal image quality was not different between myopic children undergoing AOK and OK treatments over two years. The result indicated that retinal image quality cannot account for the improved effect in retarding axial elongation resulted from the combination treatment. However, reduced VSOTF based on HOA only and less axial elongation in the AOK group, could point to a potential visual signal related to HOA that may slow eye growth.

## Data Availability

The data supporting the findings of this study are available within the article, and supplementary material can be shared upon request.
